# Intestinal Obstruction from Charcoal

**Published:** 1885-07

**Authors:** 


### Intestinal Obstruction From Charcoal.

A patient who was lately under my care, suffering from chronic obstruction in the intestinal canal, probably in the descending colon, took, on his own account, freely of charcoal powder, often a drachm a day, to relieve flatulency. The result was an all but fatal obstruction. Fortunately, under the use of enemata, coupled with persistent but gentle abdominal friction, relief was obtained, although stercoraceous vomiting once occurred. The excretions causing the obstruction were coated with carbon, and portions of carbon also passed in the free state. This is the second case in my practice in which charcoal powder has caused intestinal obstruction.—*Asclepiad, Oct.*, 1884.

